# Signalling, Metabolic Pathways and Iron Homeostasis in Endothelial Cells in Health, Atherosclerosis and Alzheimer’s Disease

**DOI:** 10.3390/cells9092055

**Published:** 2020-09-08

**Authors:** Emy Bosseboeuf, Claudio Raimondi

**Affiliations:** William Harvey Research Institute, Barts and The London School of Medicine and Dentistry, Queen Mary University of London, Charterhouse Square, London EC1M 6BQ, UK; e.bosseboeuf@qmul.ac.uk

**Keywords:** endothelial cells, angiogenesis, homeostasis, endothelial metabolism, mitochondria, iron homeostasis, atherosclerosis, Alzheimer’s disease, neuropilin-1, ABCB8

## Abstract

Endothelial cells drive the formation of new blood vessels in physiological and pathological contexts such as embryonic development, wound healing, cancer and ocular diseases. Once formed, all vessels of the vasculature system present an endothelial monolayer (the endothelium), lining the luminal wall of the vessels, that regulates gas and nutrient exchange between the circulating blood and tissues, contributing to maintaining tissue and vascular homeostasis. To perform their functions, endothelial cells integrate signalling pathways promoted by growth factors, cytokines, extracellular matrix components and signals from mechanosensory complexes sensing the blood flow. New evidence shows that endothelial cells rely on specific metabolic pathways for distinct cellular functions and that the integration of signalling and metabolic pathways regulates endothelial-dependent processes such as angiogenesis and vascular homeostasis. In this review, we provide an overview of endothelial functions and the recent advances in understanding the role of endothelial signalling and metabolism in physiological processes such as angiogenesis and vascular homeostasis and vascular diseases. Also, we focus on the signalling pathways promoted by the transmembrane protein Neuropilin-1 (NRP1) in endothelial cells, its recently discovered role in regulating mitochondrial function and iron homeostasis and the role of mitochondrial dysfunction and iron in atherosclerosis and neurodegenerative diseases.

## 1. Introduction

Endothelial cells (ECs) line the lumen of all vessels of the vascular systems and form the endocardium. In the early stages of development, proliferation and migration of ECs are essential to promote vascular morphogenesis and growth. Once the vessels are formed, ECs constitute a quiescent monolayer, selectively permeable, and rarely proliferate over months or years [1]. However, ECs retain proliferative capacity since disruption of the continuity of the endothelial monolayer stimulates ECs proliferation and migration to restore the monolayer integrity [2,3]. The plethora of signalling pathways and mechanisms regulating endothelial homeostasis is essential to form the vascular system during embryonic development and to preserve the endothelial function required for vascular and tissue homeostasis during the lifetime of an organism.

This review provides an overview of the physiological functions of the endothelium and outlines the molecular pathways regulating physiological endothelial processes such as angiogenesis, response to the shear force and the role of the endothelium in iron homeostasis. Also, we will review recent advances showing the role of metabolic pathways in regulating endothelial function and explore how signalling and metabolic pathways intertwine to regulate EC function and homeostasis. To this end, we will focus on the role of Neuropilin-1 (NRP1) as a key signalling hub and its recently described role in regulating mitochondrial dynamics, function, and iron homeostasis via the mitochondrial transporter ABCB8 in ECs. Finally, we will discuss the impact of endothelial dysfunction in both vascular disease (atherosclerosis) and non-vascular disease (Alzheimer’s Disease; AD), and investigate the holistic role of EC in the body by reviewing the potential relationship between atherosclerosis and AD.

### 1.1. Overview of the Endothelial Function

The endothelial monolayer lining the blood vessels tightly regulates the exchange of nutrients between the blood and the surrounding tissues. In addition, a healthy endothelium has anti-coagulant and anti-thrombotic properties elicited by the expression of anti-coagulant molecules such as Tissue Factor Pathway Inhibitor (TFPI), Endothelial Protein C Receptor (EPCR) and heparin-like proteoglycans. TFPI inhibits the coagulation cascade by direct inhibition of coagulation factors whilst EPCR promotes the activation of the protein C/protein S pathway downstream of the endothelial receptor thrombomodulin required for the assembly of the anti-coagulant complex [4,5]. In addition, ECs produce Von Willebrand Factor (VWF), a multifunctional adhesive plasma glycoprotein stored by ECs in endothelial-specific secretory organelles named Weibel Palade bodies, that is secreted in the subendothelial matrix and blood plasma. Although VWF has multiple functions, it regulates haemostasis by acting as a carrier for the blood-clotting protein Factor VIII and by mediating platelet adhesion [6,7].

The endothelium is a key regulator of vascular tone as ECs are the main biosynthetic source of the vasoactive substances nitric oxide (NO), which promotes vasodilation [8,9]. Furthermore, NO together with EC-derived prostaglandin-2 (PGI2) contributes to inhibiting platelets activation and aggregation, thus acting as a key mediator for the anti-thrombotic activity of the endothelium [10].

As the endothelium is exposed to the circulating blood, ECs interface with leukocytes and regulate their recruitment and transmigration across the vessels from the circulation to inflamed tissue. The healthy endothelium has anti-inflammatory properties and expresses low levels of pro-inflammatory cytokines such as Tumour Necrosis Factor-alpha (TNF-α), Interleukin-6 (IL-6), and adhesion molecules such as Vascular Cell Adhesion Molecule-1 (VCAM-1), Inter-Cellular Adhesion Molecule-1 (ICAM-1) and E-Selectin which promote leukocytes adhesion [11]. Endothelial activation and endothelial dysfunction, which are associated with cardiovascular diseases and severe viral infections, radically change the anti-inflammatory characteristics of the endothelium towards a pro-inflammatory phenotype by increasing the expression of VCAM-1, ICAM-1 and E-selectin, thus enhancing leukocyte adhesion and transmigration [12]. Activated ECs also produce pro-inflammatory cytokines such as Interleukins (ILs), Colony-Stimulating Factors (CSF), Granulocyte-CSF (G-CSF), Macrophage CSF (M-CSF) [13] and change from an anti-thrombotic to a pro-thrombotic phenotype [5].

### 1.2. Endothelial Response to Flow

The endothelium is anchored to a basement membrane formed by extra-cellular matrix (ECM) components such as collagen, elastin, fibronectin and laminin [14]. As blood flows within the vessels with a pulsatile flow induced by the heartbeat, ECs are exposed to shear forces. Unidirectional pulsatile high-shear flow occurs in straight unbranched vessel regions, whereas bifurcation, branches and high curvature of the vascular tree present multidirectional, disturbed flow [15,16]. Flow patterns modulate gene expression and function in ECs [17]. Laminar flow (unidirectional flow) increases the levels of the protective transcription factor Krüppel-Like Factor 2 (KLF-2) [18], inhibits Nuclear Factor kappa-light-chain-enhancer of activated B cells (NF-κB) and reduces the expression of pro-adhesion molecules such as ICAM-1, VCAM-1 and E-selectin [19], thus, inhibiting adhesion of immune cells onto ECs. Conversely, oscillatory/disturbed flow (multidirectional flow) increases Hypoxia Induced Factor (HIF)1α expression via NAPDH Oxidase (NOX)4-mediated Reactive Oxygen Species (ROS) production and decreases KLF-2 expression. The reduction of KLF-2 and the increase in HIF1α expression promote NF-κB activity, through the phosphorylation and nuclear translocation of p65 [19,20,21].

With its anti-inflammatory and pro-homeostatic signals, laminar flow prevents EC activation and promotes endothelial function, while disturbed flow primes ECs toward a pro-inflammatory phenotype contributing to the onset and progression of vascular diseases such as atherosclerosis [15,16]. Importantly, disturbed flow has been shown to induce EC senescence, and atherosclerotic lesions present senescent ECs which likely contribute to disease progression by creating a pro-inflammatory and a pro-thrombotic environment [22,23,24].

### 1.3. Flow Mechanosensors in Endothelial Cells

ECs are able to sense the magnitude and directionality of the shear forces and to respond to shear stress through the activation of mechanosensitive signal transduction pathways, which modulate EC morphology and function [25,26,27,28,29,30,31]. The ability of ECs to respond to flow is essential for embryonic vascular development [32,33] (see Section 2.1), vascular homeostasis in adults and vascular remodelling [20]. For instance, the redistribution of pressure following an acute artery occlusion increases the blood flow in collateral vessels and the shear stress applied on the endothelium. High level of shear stress in collateral vessels induces vessel diameter growth (arteriogenesis), stimulating cellular proliferation and cytoskeletal rearrangement in ECs [34,35]. Mechanosensors, such as integrins, tyrosine kinase receptors, G-proteins and G protein-coupled receptors, ions channels and intercellular junction proteins, are involved in sensing the shear stress induced by flow and triggering a cellular signalling response. For example, in HUVECs, shear stress promotes integrin α5β1 and ανβ3 binding to fibronectin and vitronectin (ECM proteins) [36], inducing integrin ανβ3 association with Shc and the subsequent activation of JNK. Additionally, in bovine aortic ECs (BAECs) under flow condition, binding of integrin ανβ3 to vitronectin increases ERK kinase and ERK activity [37], opening non-selective cation channel located at the plasma membrane and increasing the intracellular Ca^2+^ concentration transient [38] leading to the retractation of spontaneous migrating BAECs [39]. Furthermore, Ca^2+^ channel ATP-gated P2X4 increases intracellular Ca^2+^ in a flow-dependent manner in the presence of extracellular ATP [38], thus decreasing eNOS activity and increasing IL-6 levels [40]. EC-specific P2rx4 (gene coding for P2X4) knock-out mice show that flow-depend NO production is mediated by P2X4, impairing the flow-induced ERK1/2 phosphorylation and disrupting the vascular tone and vascular remodelling [36]. In addition to integrins, a mechanosensory complex, consisting of Platelet Endothelial Cell Adhesion Molecule 1 (PECAM-1), VE-Cadherin and Vascular Endothelial Growth Factor Receptor (VEGFR) 2 [41] is located at the cell–cell junctions. In response to flow, this mechanosensory complex induces ligand-independent phosphorylation of VEGFR2 and integrin-mediated cytoskeletal remodelling to promote cytoskeletal actin fibers alignment to the direction of flow [42,43]. In response to flow, integrins and VEGFR2 interact with the adaptor protein Shc, whose phosphorylation promotes NF-κB activity, enhancing the nuclear translocation of p65 and leading to the increase of VCAM-1 expression and leukocyte adhesion [42,43]. The signalling promoted by the mechanosensory complex through Shc stimulates arteriogenesis and improves perfusion recovery following ischemia by mediating vessel inflammation as well as proliferation, both of which are critical for arteriogenesis [43]. In addition, in this context, Shc promotes Notch-dependent expression in ECs of the arterial marker ephrinB2 [43].

In physiological conditions, activation of the mechanosensory complex promotes an inflammatory response, which is transient in ECs exposed to laminar flow and sustained in ECs exposed to disturbed flow [20,31]. Recent evidence has shown that VEGFR3 plays a role in the endothelial response to flow [44] and that intramembrane binding of VE-Cadherin to VEGFR2 and VEGFR3 is required to assemble the endothelial mechanosensory complex [41]. VEGFR3 is highly expressed in lymphatic and comparatively less expressed in angiogenic ECs [44]. Also, VEGFR3 is expressed by aortic ECs in the inner curvature of adult mouse aortas while its expression is weaker in ECs from other aortic regions [41]. In both lymphatic and blood ECs, VEGFR3 expression levels determine EC sensitivity to flow. Accordingly, reducing VEGFR3 expression in lymphatic ECs increases the level of shear stress required to trigger a flow-induced response, while increased VEGFR3 in blood vessel ECs decreases the shear force required to elicit a response [44].

Thus, in established vessels the response of ECs to flow plays an integral part to modulate endothelial function. Importantly, hemodynamic force also regulates vascular remodelling after vasculogenesis during embryonic development (embryonic vascular development is discussed in Section 2). Accordingly, knockout mouse mutant lacking the expression of atrial myosin light chain 2 or sodium calcium exchanger 1, two heart-specific proteins required for heart function, have impaired vascular development caused by the reduced hemodynamic force.

## 2. Angiogenesis in Development and Diseases

### 2.1. Drivers of Angiogenesis

The cardiovascular system is formed early during embryonic development. Once the embryo reaches a volume of 1–2 mm^3^, the diffusion of nutrients and oxygen (O_2_) is limited and the consequent hypoxia stimulates the formation of a vascular network [45]. The first embryonic vessel is formed by coalescing angioblasts, which rise in the mesoderm and assemble a primitive tubular network in a process called vasculogenesis [46,47,48]. Thereafter, new vessels sprout from pre-existing ones in a process termed angiogenesis [49,50]. Thus, vasculogenesis and angiogenesis contribute to the morphogenesis of the vascular tree formed by arteries, veins and capillaries.

**Figure 1:**
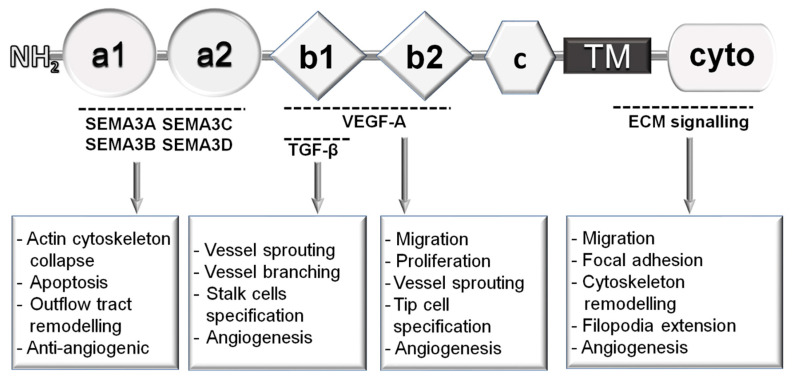
Representation of the transmembrane protein Neuropilin-1 (NRP1): NRP1 consists of seven domains, two complement (CUB) domains (a1 and a2), two coagulation factor (FV/FVIII) domains (b1 and b2), a MAM domain with homology to the meprin/antigen 5/receptor tyrosine phosphatase μ domain (c), a transmembrane domain (TM) and a cytoplasmic domain (cyto) that interacts with intracellular proteins. The boxes indicate the signalling pathways promoted by the binding of endothelial NRP1 to the indicated ligands.

In hypoxic conditions, the availability of O_2_ is not sufficient for the metabolic need of cells and tissues. Cells exposed to hypoxia up-regulate the expression of transcription factors belonging to the HIF family [51]. The HIFs-dependent hypoxia response induces the secretion of several pro-angiogenic factors such as Vascular Endothelial Growth Factor-A (VEGF-A), Platelet-derived Growth Factor (PDGF) and Angiopoietin-2 stimulating angiogenesis to increase oxygen perfusion [52,53], [54]. VEGF-A is one of the most potent and extensively studied pro-angiogenic factors. In ECs, it interacts with the transmembrane tyrosine kinase receptor VEGFR2 and its co-receptor Neuropilin-1 (NRP1) to promote downstream signals [55,56] (Figure 1).

VEGF-A also interacts with tyrosine kinase receptor VEGFR1, whose signalling role in ECs is not completely understood and that is conventionally described as a decoy receptor in angiogenesis, preventing VEGF-A binding to VEGFR2 [57,58].

VEGF-A binding to its receptor promotes phosphorylation of p130cas, paxillin, p38 Mitogen-Activated Protein Kinase (MAPK) and the activation of small GTPases [59,60,61,62], thus promoting endothelial motility. Furthermore, activation of VEGFR2 promotes cell proliferation and survival by activating Extracellular signal-Regulated Kinase 1/2 (ERK1/2) [63,64] and Phosphoinositide-3 Kinases (PI3Ks), which leads to the activation of AKT [65,66]. Thus, VEGF-A-dependent signalling pathways are essential to ensure directional migration and increased proliferation, which are both required for sprouting angiogenesis.

### 2.2. Sprouting in Angiogenesis

Sprouting angiogenesis has been extensively investigated in the zebrafish larval trunk, the mouse embryo hindbrain and the mouse retina. In the retina, the preformed astrocyte network produces a VEGF-A gradient towards the retinal periphery [67]. Following the VEGF-A gradient, endothelial sprouts migrate from the optic disc toward the peripheral margin on a network of astrocytes and are guided by astrocyte-derived fibronectin, which activates integrin signalling in ECs, providing further pro-angiogenic stimuli and directional cues [67,68,69,70]. Connections between migrating sprouts occur when tip cells fuse in a process called anastomosis to form a perfused vascular network. Then, the vessels undergo remodelling, followed by recruitment of pericytes, and an overall reduction of pro-angiogenic stimuli, leading to the vessel maturation [49,71,72]. During angiogenesis, endothelial sprouts are headed by filopodia-studded ECs in tip position (tip cells), with high migratory and low proliferative capacity, which migrate towards a VEGF-A gradient. Behind the tip cells, ECs with high proliferative capacity assume a stalk position (hence named stalk cells) and support the growth of the new vessel by forming the wall of the sprout and the nascent vascular lumen [73,74]. Specification of tip and stalk ECs is a dynamic process in which VEGF-A induces expression of the transmembrane ligand Delta-like ligand-4 (DLL4) in tip cells, which binds to the Notch receptors in adjacent ECs. The Dll4/Notch signalling activated in the ECs adjacent to the tip cell inhibits the tip phenotype via a lateral inhibition mechanism and modulates gene expression defining a stalk phenotype [75,76].

### 2.3. VEGF as a Therapeutic Target in Pathological Angiogenesis

VEGF-A has been extensively studied in angiogenesis because it is essential for all stages of cardiovascular development and plays a major role in tumour angiogenesis and neovascularisation in eye pathologies such as Age-related Macular Degeneration (AMD) and Proliferative Diabetic Retinopathy (PDR) [74,77,78]. In addition to promoting angiogenesis, VEGF-A regulates vascular permeability [79]. Pathological upregulation of VEGF-A levels and signalling induces hyperpermeability that causes tissue injury and oedema [80,81]. The depletion of the gene encoding for VEGF-A or encoding its tyrosine kinase receptor KDR in experimental mice results in embryonic lethality [82,83,84,85]. In pathological conditions such as eye diseases and tumours, VEGF-A is overexpressed and the resulting neovascularisation promotes the formation of immature and leaky vessels, causing tissue oedema. Accordingly, anti-VEGF therapy is the approved treatment for AMD and PDR and for some solid tumours such as colorectal and lung cancers, glioblastoma, renal cancer, hepatocellular carcinoma and gastrointestinal stromal tumours [86]. In the case of AMD, anti-VEGF therapy stabilises the sight in over 90% of cases, although only 30% of people show improved vision [87]. Importantly, 5-years and 7-years follow-up studies have shown that long-term anti-VEGF monotherapy increases the risk of developing geographic atrophy, a form of chronic progressive degeneration of the macula [87,88,89]. In cancer, anti-VEGF therapy is effective on a subset of metastatic tumours but overall has shown modest results than predicted, failing to show significant effects in advanced-stage tumours and being effective mainly when combined with chemotherapy [90,91]. This is likely due to the developing of resistance mechanisms in the tumour environment which upregulate other pro-angiogenic factors capable of maintaining an active pro-angiogenic environment such as Bone Morphogenetic Protein (BMP) and basic Fibroblast Growth Factor 2 (bFGF2) [92,93].

Conversely, injections of VEGF-A could help stroke recovery by promoting the neovascularisation of the ischemic region [94]. After stroke events VEGF-A, VEGFR1 and VEGFR2 expression are naturally increased [95,96,97]. Animal studies of rats middle cerebral artery occlusion (MCAO) model have shown that VEGF-A intravenous injections 1 day after the event had a beneficial effect on the recovery [98,99,100], whereas injections within 24 hrs after the event had detrimental effects [100,101].

In hypoxic regions, VEGF-A is secreted triggering ECs angiogenesis and guiding endothelial sprouts. As VEGF-A is one of the most potent pro-angiogenic factors together with vascular permeability, elucidating VEGF-A-mediated signalling transduction has been the focus of many groups in the field. To activate downstream signalling pathways VEGF-A binds to its tyrosine kinase receptor VEGFR2 and to the transmembrane protein Neuropilin-1 (NRP1). The role of NRP1 in VEGF-dependent and -independent signalling is discussed in Section 3.

## 3. Neuropilin-1 Signalling in Endothelial Cells

Neuropilin-1 (NRP1) is a 134 kDa transmembrane receptor expressed in the placenta, brain, heart, kidney and the vascular system [55]. Mice lacking NRP1 expression die before birth because of defects in cardiovascular and neuronal development and the endothelial-specific NRP1 knockout mice recapitulate the defects of the global knockout [102,103,104,105]. Accordingly, several studies have shown the involvement of NRP1 in the development of the embryonic vasculature and lymphatic valve [106,107,108].

### 3.1. Neuropilin-1-Dependent Semaphorin Signallings

NRP1 is known to be a co-receptor for members of the Semaphorin-3 (SEMA3) family and VEGF-A in neurons and ECs. NRP1 binds SEMA3A SEMA3B, SEMA3C and SEMA3D via its a1 and a2 domains (Figure 1) and forms a holoreceptor by interacting with members of the transmembrane receptors plexins (PLXNs) such as PLXND1 and PLXNA4 [109]. Mouse mutants expressing a form of NRP1 with a point mutation in the a1 domain abrogating SEMA3A binding, show defects in axonal pathfinding, in addition to cardiac and lung vascular abnormalities [110,111,112]. In EC, binding of SEMA3B to NRP1 has anti-angiogenic activity by collapsing the actin cytoskeleton and inhibits VEGF-A signalling resulting in apoptosis and reduced angiogenesis [113] (Figure 1). Similarly, SEMA3C inhibits angiogenesis [114] by binding the holoreceptor formed by NRP1 and PLXND1 and promotes endothelial-to-mesenchymal transition during the embryonic development of the outflow tract in a process essential to form the endocardial cushions [115]. Finally, binding of SEMA3D to NRP1 mediates EC repulsion and pulmonary vein patterning during embryogenesis via a PI3K/AKT pathway which modulates cytoskeleton remodelling endothelial migration and guidance [116] (Figure 1).

### 3.2. Role of Neuropilin-1 in VEGF Signalling

NRP1 binds VEGF-A via the b1 and b2 domains [117] (Figure 1). While the b1 domain is essential to bind VEGF-A as deletion of this domain abrogates VEGF-A binding to NRP1, loss of the b2 domain only reduces NRP1 affinity to VEGF-A [118]. Several groups have shown that binding of VEGF-A to NRP1 potentiates VEGF-A-mediated signalling in ECs. Co-expression of NRP1 and VEGFR2 in porcine aortic ECs increases VEGF-A-induced migration and the phosphorylation of VEGF-A downstream signalling effectors such as AKT, ERK1/2 and p38 MAPK kinases compared to cells expressing VEGFR2 only [55,56]. Conversely, blocking NRP1 binding to VEGF-A with anti-NRP1 blocking antibodies abrogating VEGF-A binding to NRP1 but not to VEGFR2, reduces VEGF-induced EC migration, proliferation, vessel sprouting and neovascularisation in the eye, although to a lesser extent compared to VEGF blocking antibodies [65]. Furthermore, treatment with anti-NRP1 blocking antibodies preventing NRP1 binding to VEGF-A, partially decreases VEGF-A-induced EC proliferation and AKT, ERK1/2 and p38 MAPK phosphorylation compared to treatment with an anti-VEGF-A antibody which completely abrogated these VEGF-A-induced responses [65] (Figure 1).

Although these data support the role of NRP1 as a positive modulator of VEGF-A signalling, the fact that VEGF-A binding to NRP1 is dispensable for vascular permeability in vivo and that combining anti-NRP1 with anti-VEGF-A blocking antibodies has a synergistic effect on tumour angiogenesis and growth, suggests that NRP1 also promotes VEGF-A independent signalling [65]. Accordingly, a bi-specific antibody generated by genetic fusion of the C-terminus of the anti-VEGF-A antibody Bevacizumab with a peptide that specifically binds to the VEGF-binding pocket in NRP1 b1 domain [119], inhibits EC migration induced by pro-angiogenic factors and has a more potent anti-tumour activity than Bevacizumab in a murine tumour xenograft model [120].

### 3.3. VEGF-Independent Role of Neuropilin-1 in Angiogenesis

Mutations in NRP1 b1 domain of tyrosine in position 297 into alanine (Y297A) or aspartic acid in position 320 into alanine (D320A) abrogate NRP1 binding to VEGF-A and reduces endothelial migration in vitro [121]. Generation of *Nrp1^Y297A^* mouse mutants revealed that VEGF-A binding to NRP1 is not essential for embryonic angiogenesis [122] and mutants were born at normal Mendelian ratios. Importantly, *Nrp1^Y297A^* mutants showed reduced hindbrain, retinal and tumour angiogenesis. However, the gene-targeting strategy to generate the *Nrp1^Y297A^* mouse mutant resulted in a reduction of NRP1 expression, generating a NRP1 hypomorph. Thus, the phenotype observed results from the combination of reduced NRP1 expression and its inability to bind VEGF-A. Gelfand and colleagues generated a *Nrp1^D320A^* mouse mutant, which has normal NRP1 levels but impaired VEGF-A binding to NRP1. *Nrp1^D320A^* mouse mutants are born at the expected Mendelian ratio, have no gross embryonic vascular or cardiac phenotypes and show normal cortical vessel branching and coverage in the brain [123]. However, NRP1*^D320A^* mutants show delayed postnatal angiogenesis and a reduction in the number of arteries in the retina [123]. Although the retinal plexus of adult NRP1*^D320A^* have similar coverage to that of littermate controls, adult NRP1*^D320A^* have consistently lower arteries. Importantly, in a model of hind-limb ischemia, these mutants show reduced post-ischemic arteriogenesis [123], similarly to mice lacking the NRP1 cytoplasmic domain [124]. Thus, although NRP1 promotes VEGF-A-mediate response and signalling which regulates some aspects of vascular development and postnatal arteriogenesis, NRP1-mediated VEGF signalling is dispensable for developmental angiogenesis. As endothelial-specific deletion of NRP1 results in severe angiogenic defects, NRP1 likely promotes angiogenesis via VEGF-independent mechanisms.

### 3.4. Role of Neuropilin-1 in Integrin and TGFβ-Mediated Signals

NRP1 has been reported to modulate integrin signalling and extracellular matrix remodelling in ECs and tumours (Figure 1). In ECs, following stimulation with the extracellular matrix component fibronectin, NRP1 forms a complex with activated α5β1 integrin at the plasma membrane at the level of the adhesion sites. NRP1 stimulates Rab5/Rab21-dependent internalisation of active α5β1 integrin into endosomes to promote integrin signalling [125]. In tumours, NRP1 promotes integrin α5β1 fibronectin fibril assembly activity and desmoplasia by favouring the interaction between the non-receptor tyrosine kinase ABL1 and the scaffolding protein GIPC [126]. In agreement with a role of NRP1 in integrin activation and signalling, NRP1 mediates EC adhesion to fibronectin independently of VEGFR2 [127] and promotes fibronectin-induced EC migration [70] through a pathway that promotes ABL1 kinase activation [70] (Figure 1). The NRP1-dependent activation of ABL1 leads, on one hand, to the phosphorylation in residue Y118 of the focal adhesion component paxillin [70], which is required for focal adhesion maturation and turnover [128,129] and, on the other hand to the activation of the small Rho-GTPases CDC42, regulating cytoskeleton remodelling and filopodia extension [130]. The NRP1-ABL1 pathway has a role in physiological angiogenesis in vivo as shown by the observation that the phenotype of NRP1 endothelial-specific knockout, which show fewer tip cells and branchpoint in the retinal plexus, is phenocopied in mice treated with ABL1 or CDC42 inhibitors [70,130]. Similarly, treatment with the ABL1 inhibitor imatinib reduced growth of abnormal vessels in a mouse model of pathological angiogenesis [70].

Several studies have also shown that NRP1 is able to modulate the TGFβ pathway in different contexts and that NRP1 works as a signalling hub integrating VEGF-A, integrin and TGFβ signalling (Figure 1). Latent and active TGFβ compete with VEGF-A to bind NRP1 via the b1 domain and NRP1 promotes TGFβ ligand activation in a mechanism requiring the b2 domain [131]. Furthermore, NRP1 interacts with TGFβ receptor type 1 (e.g., ALK1 and ALK5) and the TGFβ receptor 2 (TGFBR2), independently of TGFβ binding and act as a TGF-β co-receptor in breast cancer cell lines augmenting canonical SMAD2/3 signalling [132]. During brain development, the NRP1 expressed in neuroepithelial cells promotes trans-interaction between endothelial NRP1 and neuroepithelial ανβ8 integrin, suppressing the integrin β8-dependent activation of the ECM-bound latent TGFβ and inhibiting the TGFβ receptors signalling in ECs [133]. Accordingly, knockout of neuroepithelial ανβ8 integrin in mice decreases SMAD3 phosphorylation in EC to similar levels of endothelial-specific knockout of TGFβR2 [133]. In agreement with a role of endothelial NRP1 in suppressing TGFβ signalling in ECs, E12.5 endothelial-specific NRP1 knockout embryos show increased SMAD3 phosphorylation in ECs of the cerebral cortices [133]. Similarly, downregulation of NRP1 in cultured ECs increases SMAD3, SMAD1/5/8 as well as ERK1/2 phosphorylation [133]. These data indicate that NRP1 suppresses paracrine and autocrine TGFβ in ECs, in stark contrast with the role of NRP1 as a promoter of TGFβ signalling in cancer cells. This difference is likely due to the cell-specific expression of NRP1 co-receptors or binding partners or to the trans-interactions of NRP1 with proteins expressed by other cell types or embedded in the ECM in a context-dependent manner, which result in diametrically opposite NRP1 functions.

The function of NRP1 as a suppressor of TGFβ signalling in ECs regulates vascular sprouting and branching during postnatal angiogenesis [134] (Figure 1). By limiting SMAD2/3 phosphorylation, NRP1 expression inhibits a stalk-cell phenotype and promotes EC competition for tip position in endothelial vascular sprouts [134]. Mechanistically, VEGF-promoted DLL4 production in tip cells which activates NOTCH signalling and decreases NRP1 expression in stalk cells, thus enhancing SMAD-dependent signalling and stalk cell behaviour [134].

**Figure 2:**
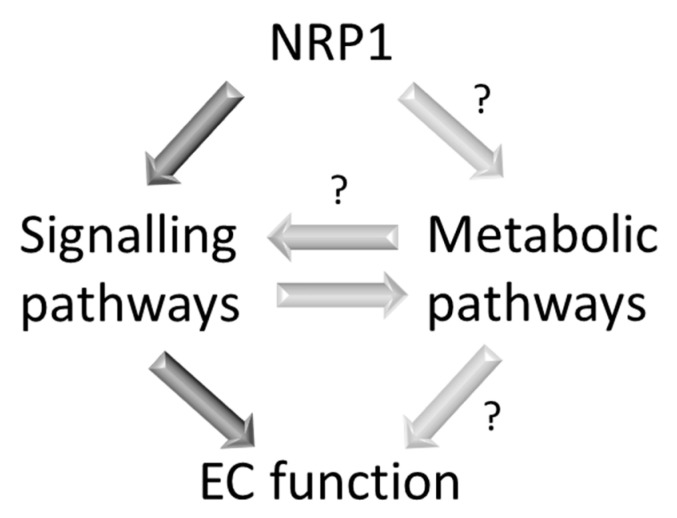
Regulatory functions of NRP1 in endothelial cells (ECs): Diagram illustrating established (grey arrows) and potential (shaded arrows) pathways by which NRP1 regulates EC function.

As seen in this chapter, NRP1, located at the plasma membrane, modulates angiogenesis through VEGF-dependent as well as VEGF-independent signalling pathways. It is now established that in addition to signalling pathways, modulation of metabolic pathways in ECs co-determines blood vessel growth (see Section 4). We recently discovered that a pool of NRP1 localises in the mitochondria and regulates mitochondrial activity by suppressing iron-dependent oxidative damage [135] (see Section 6). As mitochondria play a key role in bioenergy production and in biosynthetic pathways in ECs (discussed in Section 5), the finding that NRP1 acts as a regulator of mitochondrial homeostasis suggests that NRP1 could regulate angiogenesis and EC function also by promoting metabolic pathways, either autonomously or through the activation of signalling pathways resulting in changes in EC metabolism (Figure 2).

## 4. Metabolism and Endothelial Function

The role of signalling pathways elicited by pro-angiogenic factors and their receptors is now well understood and targeting growth factors has been the main strategy to curb pathological angiogenesis. However, anti-angiogenic therapies targeting angiogenic factors, although effective to treat some pathologies (as discussed in Section 2.3), show limited efficacy, have side effects due to some degree of systemic toxicity in patients with cancer or eye diseases and they often become ineffective because of the insurgence of resistance [90]. Recent evidence shows that ECs modulate metabolic pathways to drive angiogenesis [136] and that the manipulation of EC metabolism inhibits vessel sprouting in response to pro-angiogenic factors [137]. To meet the cellular energetic and metabolic needs, metabolism produces Adenosine Triphosphate (ATP) through the aerobic [138,139], [140] and the anaerobic pathways [141], via glycolysis and the mitochondrial respiration respectively. Furthermore, ECs rely on metabolites of the Tricarboxylic Acid (TCA) cycle for biomass production and biosynthetic pathways [142,143]. The role of these metabolic pathways in EC function is reviewed in the following Section 4.1, Section 4.2, Section 4.3 and Section 4.4.

### 4.1. Glycolytic Flux and Angiogenesis

**Figure 3:**
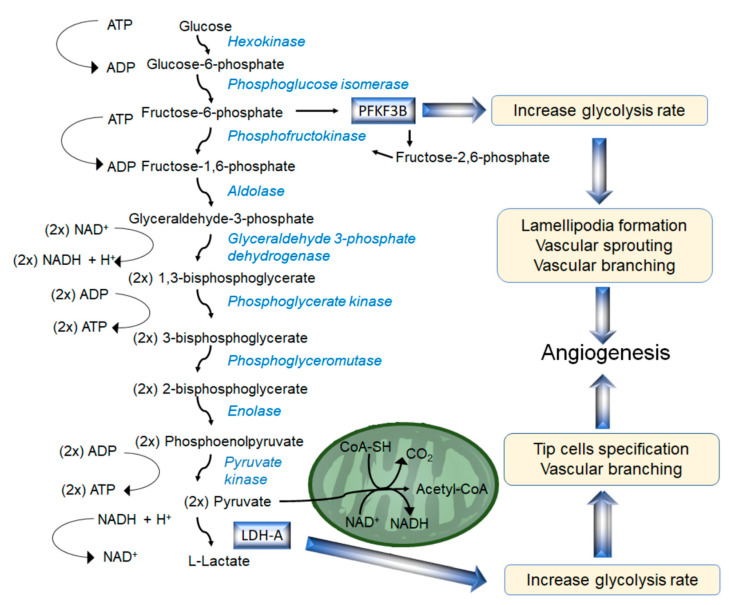
Enzymatic reactions of the glycolytic pathways: Glucose is converted to pyruvate in a series of enzymatic reactions. Pyruvate is then either transformed into L-Lactate by LDH-A (Lactate dehydrogenase) or translocated into the mitochondria and transformed into Acetyl-CoA before being integrated into the Tricarboxilic Acid (TCA) cycle. The enzymes PFKF3B (phosphofructo-2-kinase/fructose-2,6-biphosphatase) and LDH-A are part of a positive feedback loop and are upregulated by ECs to increase the glycolytic flux.

Since hypoxia is one of the main physiological drivers of angiogenesis and endothelial sprouts face low oxygen availability, ECs rely mainly on anaerobic glycolysis to produce ATP to meet their energy demand [138,144,145,146,147]. Glucose undergoes metabolic breakdown through a series of anaerobic enzymatic reactions which transform it into fructose and eventually into pyruvate (Figure 3).

**Figure 4:**
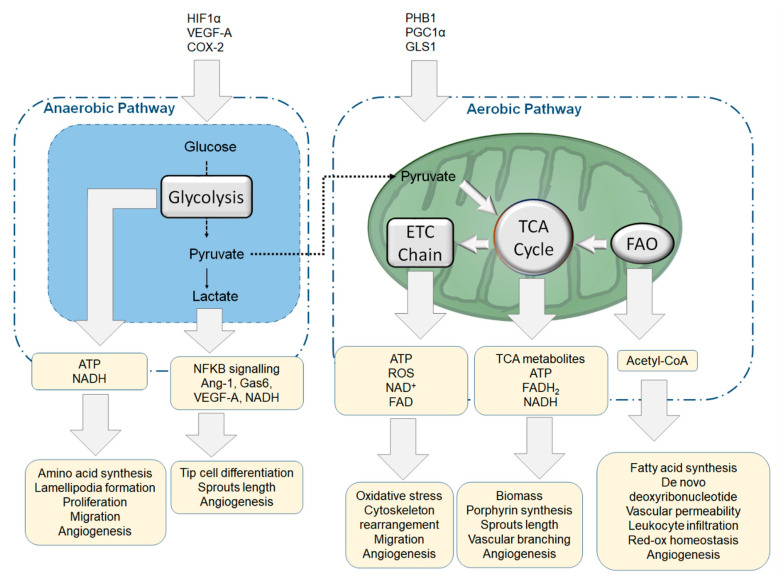
Crosstalk between signalling and metabolic pathways: The diagram illustrates the regulation of the anaerobic and aerobic metabolic pathways in ECs downstream of signalling pathways induced by cytokines (i.e., VEGF-A) or modulated by cellular proteins and enzymes (i.e., HIF1α, COX-2, PHB1, PGC1α, GLS1). Cellular products of both metabolic pathways are highlighted together with their contribution towards specific EC functions.

6-phosphofructo-2-kinase/fructose-2,6-biphosphatase (PFKFB3) is a key enzyme in regulating glycolytic flux in mammalian cells. It phosphorylates D-fructose 6-phosphate producing fructose-2,6-bisphosphate which acts as a potent positive allosteric effector of 6-phosphofructo-1-kinase (PFK-1), thus enhancing glycolysis [148]. The glycolytic metabolic pathway has a net yield of 2 moles of NADH, 2 moles of ATP and 2 moles of pyruvate per mole of metabolised glucose (Figure 3). In anaerobic conditions, lactate dehydrogenase-A (LDH-A) catalyses the anaerobic conversion of pyruvate into lactate [149]. During angiogenesis, ECs requires energy and metabolites for biomass production, migration and proliferation to form new vessels. Thus, in response to pro-angiogenic factor, ECs can increase their glucose metabolism by upregulating the expression of glucose transporter 1 and of glycolytic enzymes, such as LDH-A and PFKFB3 to increase the glycolytic flux [138,145,146,150] (Figure 3 and Figure 4).

In the tumour microenvironment, the high level of glycolysis of tumour ECs and tumour cells induces lactate production, which acts as a further pro-angiogenic stimulus [151,152]. Accordingly, in addition to being a product of EC metabolism, extracellular lactate acts as a signalling molecule and is internalised in ECs via the Monocarboxylate Transporter-1 (MCT1), increasing the expression and secretion of the growth factors Gas6, Angiopoietin-1 (Ang1) and VEGF-A. These factors activate the PI3K/AKT pathway downstream of Axl, Tie2 and VEGFR2 respectively, thus promoting tube formation and endothelial sprouting [153,154]. In ECs, lactate also increases ROS production which activates NF-κB signalling and NF-κB-dependent IL-8 production by promoting the phosphorylation and degradation of NF-κB inhibitor IκB-α. This pathway has been shown to play a major role in tumour angiogenesis since reducing lactate production from tumours, reduces the lactate/NF-κB signalling pathway in ECs and NF-κB-dependent IL-8 production, inhibiting tumour angiogenesis [155] (Figure 4).

In addition, the lactate/NF-κB signalling pathway promotes angiogenesis and neurogenesis in a rat model of intracerebral haemorrhage [156]. Since the increased glycolytic rate of tumour ECs resulting in high lactate production and excretion [137,157] activates pro-angiogenic signalling pathways in a positive feedback loop, targeting glycolysis and reducing lactate production in tumours could represent a promising therapeutic target for cancer treatment.

### 4.2. 6-Phosphofructo-2-Kinase/Fructose-2,6-Biphosphatase (PFKFB3) in Physiological and Pathological Angiogenesis

Pharmacological or genetic inhibition of PFKFB3 in ECs impairs the formation of lamellipodia and filopodia, reducing EC ability to migrate, to sprout and to branch, thus leading to defective physiological vascular development and reduced pathological angiogenesis [138,145,158,159]. Evidence suggests that PFKFB3 blockade reduces the proliferation and migration of the ECs (both in vitro and in vivo) but does not affect the expression of Dll-4, VEGFR2 and Notch1, which regulate tip/stalk specification [158,160]. However, a recent study suggests that the glycolytic anaerobic pathway and the aerobic mitochondrial respiration (discussed in Section 4.3), regulate the specification of tip and stalk cells with glycolysis regulating initial tip cell formation [161] (Figure 4). Accordingly, inhibition of LDH-A, which produces lactate from pyruvate and promotes glycolysis in ECs [146], reduces the percentage of tip cells and decreases the expression of tip cell-enriched genes such CD34, DLL4 and VEGFR2 [161]. Conversely, blocking of the aerobic pathway by inhibiting Pyruvate DeHydrogenase E1 Alpha 1 component (PDHA1) increased the fraction of tip cells and induced the differentiation of non-tip cells into tip cells together with increasing the expression levels of CD34, DLL4 and VEGFR2 [161]. Interestingly, during sprouting angiogenesis in chicken embryos, inhibition of the expression of PDHA1, PFKFB3, or LDHA similarly reduced sprout lengths and differentiation of non-tip cells into tip cells [161] while silencing PDHA1 or LDHA expression, but not PFKFB3, reduced the number of branching points. These findings indicate that glycolysis, as well as mitochondrial respiration, are essential for sprouting angiogenesis and agree with the idea that a “metabolic switch” [138,162] occurs during tip/stalk differentiation with glycolysis necessary for tip cell differentiation and with glycolysis and mitochondrial respiration essential for EC proliferation and survival [161,163].

In tumour ECs, VEGF-A and PFKFB3 play a major role in enhancing EC metabolism since inhibition of Prostaglandin endoperoxide synthase 2, also known as cyclooxygenase-2 (COX-2) decreases the expression of VEGF-A and PFKFB3 and reduces the glycolysis rate to the level of normal ECs [157]. Tumour vessels are characterised by high leakiness and by alterations in pericyte phenotype and coverage [164]. The elevated permeability of the tumour vasculature favours tumour cell intravasation in the vascular system and metastasis dissemination [164,165]. In addition to its role in angiogenesis, PFKFB3 expression in tumour vasculature plays a role in metastasis dissemination by stimulating vascular permeability. Accordingly, PFKFB3 downregulation has been shown to decrease the metastatic capacity of cancer cells by reducing vascular permeability through the tightening of VE-Cadherin-mediated endothelial junctions without affecting tumour growth [137].

### 4.3. Aerobic, Anaerobic Respiration and Mitochondria Homeostasis in ECs

In addition to be a substrate for LDH-A, pyruvate is translocated into the mitochondria and transformed into Acetyl-CoA by the pyruvate dehydrogenase, which feeds it into the Tricarboxylic Acid (TCA) cycle, known also as Krebs cycle. Metabolic intermediates of the TCA cycle are systematically oxidised to reduce NAD^+^ and FAD to NADH and FADH_2_, which subsequently will be used in the mitochondria in the process of oxidative phosphorylation as electron donors [166,167]. Mitochondrial complex I oxidizes NADH and passes electrons to the mitochondrial complexes of the Electron Transport Chain (ETC) located at the mitochondrial inner membrane, in a series of red-ox reactions. The transfer of the electrons across the ETC is coupled to the pumping of protons (H^+^) from the mitochondrial matrix to the intermembrane space, thus creating a H^+^ gradient across the mitochondrial inner membrane. The transmembrane potential of protons forms the proton motive force that provides the energy to complex V, known as ATP synthase, to synthesise ATP [167]. Since O_2_ is the final acceptor of electrons from the ETC, oxidative phosphorylation can only occur in aerobic conditions.

Although aerobic respiration generates 36 moles of ATP per mole of glucose, it is slower than glycolysis which is, therefore, more suitable to quickly provide energy to highly proliferative cells [140]. ECs limitedly rely on the aerobic pathway and meet their energy demand primarily via anaerobic glycolysis [138]. Accordingly, ECs possess a low mitochondria content (2–6% of cell volume) compared to other cell types such as cardiomyocytes (32% of cell volume) [168,169]. The dependence of EC from anaerobic glycolysis for biomass and energy production is consistent with the ability of ECs to thrive and function in hypoxic conditions such as those encountered during developmental angiogenesis or neo-angiogenesis in ischemic tissues. Yet, mitochondria are essential for ECs function (Figure 4) as the alteration of mitochondria dynamics contributes to endothelial dysfunction [141]. For instance, HUVECs overloaded with iron present reduced mitochondrial maximal respiration and spare respiration capacity, which are improved using an iron chelator deferiprone [170]. Interestingly, deferiprone reduced the iron-induced increase of mitochondrial Ca^2+^ level and rescues the ROS production, suggesting a link between ECs iron homeostasis and mitochondrial respiration, Ca^2+^ and ROS production [170]. Recent studies show that mitochondria play a critical role in regulating Ca^2+^ signalling in the endothelium of isolated blood vessels [171,172,173]. Mitochondria have been shown to modulate (IP3)-mediated Ca^2+^ signalling in ECs predominated near EC-smooth muscle cells contact sites in isolated blood vessels, in a mechanism mediated by the mitochondrial membrane potential and ATP production [172].

Moreover, deletion in ECs of Prohibitin-1 (PHB1), which is highly expressed by ECs and localised in the inner membrane of mitochondria, reduces mitochondrial function by impairing ETC complex I function and promotes reactive oxygen species (ROS production, see Section 5), leading to ROS-induced senescence [174]. Furthermore, PHB1 knockdown has been reported to increase the activity of AKT and RAC1, leading to cytoskeletal rearrangement and impairing EC migration and angiogenesis [174]. Mitochondrial DNA damage correlates with the extent of atherosclerosis in patients and mouse models of atherosclerosis [175]. Furthermore, depletion of mitochondrial biogenesis regulator Peroxisome Proliferator-activated Receptor γ Coactivator 1α (PGC1α) results in vascular dysfunction and inflammation because of increased mitochondrial ROS production in response to chronic angiotensin-II infusion [176]. Since preventing mitochondrial dysfunction delays replicative senescence in human primary cells [177] and the inhibition of mitochondrial antioxidant enzymes accelerates mitochondrial damage and atherogenesis [175], promoting mitochondrial function or targeting mitochondria dysfunction could be potential therapeutic targets in atherosclerosis and cardiovascular diseases.

### 4.4. Biosynthetic Pathways and Anaplerosis Modulate Endothelial Function

Recent evidence shows that in ECs, intermediate metabolites of the TCA cycle are used as a substrate to synthesise fatty acids, amino acids and porphyrins [142] (Figure 3). Thus, anaplerotic reactions, aiming to replenish the TCA intermediates used for biosynthetic pathways, are essential for EC function [143,178]. For instance, Fatty Acid β-Oxidation (FAO) contributes to anaplerosis through the mitochondrial internalisation and metabolism of fatty acids. During this process, Carnitine Palmitoyl-Transferase I (CPT1) allows the mitochondrial internalisation of Acyl-CoA by catalysing the formation of Acyl-carnitine from Acyl-CoA and carnitine. Once in the mitochondrial matrix, CPT2 metabolises Acyl-carnitine back into Acyl-CoA, which is then used to produce Acetyl-CoA [179]. Accordingly, CPT1 inhibition prevents TCA replenishment, negatively affecting endothelial angiogenesis [143]. Although CPT1 does not interfere with filopodia formation, ADP/ATP ratio, redox balance or protein synthesis, CPT1 deletion reduces the length of cellular sprouting as well as the vascular branching, by reducing the de novo deoxyribonucleotide synthesis of nucleotides [143]. Similarly, Fatty Acid Synthase (FASN), which mediates the synthesis of palmitate from Acetyl-CoA and malonyl-CoA in the presence of NADPH [180], plays a role in angiogenesis [181,182]. FASN downregulation in EC induces accumulation of malonyl-CoA levels, which leads to malonylation of mammalian Target of Rapamycin Complex-1 (mTORC1) and subsequent inactivation of mTOR signalling pathway [182], inhibiting angiogenesis. Thus, the inhibition of FASN reduces pathological ocular neovascularisation by inhibiting mTOR activity [182]. A recent study, investigating metabolic differences between proliferative and quiescent ECs, has shown that quiescent ECs increase FAO to sustain the TCA-cycle flux to a larger extent than proliferative ECs and that although FAO is dispensable for energy homeostasis, biomass synthesis, and histone acetylation in quiescent EC, it is required for redox homeostasis through NADPH regeneration [183]. Accordingly, impairment of FAO induces EC dysfunction promoting leukocytes infiltration and permeability [183].

Glutamine metabolism in ECs contributes to replenishing α-Ketoglutarate, which is used for fatty acid or nucleotide biosynthesis [184]. Glutamine depletion or genetic ablation of glutaminase 1 (GLS1), the resident mitochondrial enzyme converting glutamine to glutamate, impairs biomass synthesis and EC proliferation leading to vessel-sprouting defects [184,185]. Similarly, Glutamine-dependent asparagine synthesis is indispensable for the growth of ECs [185]. Thus, the increasing evidence that metabolic pathways, relying partly on mitochondrial resident enzymes or metabolites for biosynthesis, are important to regulate physiological and pathological angiogenesis, highlights the role of mitochondria as important metabolic hubs whose function is essential for endothelial function. Furthermore, as endothelial dysfunction and unbalanced endothelial metabolism occur in several pathologies such as pulmonary arterial hypertension, neovascular ocular diseases and tumoral progression [91], metabolism could represent a potential target for treating endothelial dysfunction and vascular pathologies. Unbalanced metabolism and dysregulation of mitochondrial respiration can also lead to the production of mitochondrial ROS, whose excessive production leads to cytotoxic effect [186]. The role of ROS in endothelial function is reviewed in Section 5.

## 5. Reactive Oxygen Species (ROS)

ROS consist of reactive chemical species containing at least one atom of oxygen with higher reactivity than the molecular oxygen. ROS include the free radical species such as superoxide, hydroxyl radical and singlet oxygen and the non-radical hydrogen peroxide [187].

### 5.1. Mitochondrial ROS Production and Detoxification

ROS are a natural by-product of catabolic and oxidative activities and are produced in several cellular compartments such as the endoplasmic reticulum, cytosol and mitochondria [186]. Mitochondria-derived ROS (mROS) are generated as a by-product of mitochondrial respiration (Figure 4); as electrons move along the components of the ETC, a small number of electrons “leak”, reducing oxygen prematurely and generating superoxide anion (O_2_^−^) [188,189]. Complex I has been shown to produce and release O_2_^−^ in the mitochondrial matrix, probably at the level of the iron-sulphur (Fe-S) clusters contained in the matrix-protruding hydrophilic arm [190], while complex III releases O_2_^−^ into the matrix and the intermembrane space [190]. Because elevated O_2_^−^ levels induce cellular damage, the scavenger enzymes Superoxide Dismutase 1 and 2 (SOD1 and SOD2) catalyse the dismutation of O_2_^−^ to H_2_O_2_ and O_2_. Since H_2_O_2_ induces oxidative stress and acidification of the cytosol, it is converted by catalase (CAT) and Glutathione Peroxidase (GPx) into H_2_O and O_2_ [191]. Similarly, ROS is generated by the activity of NADPH Oxidase enzymes (NOXs). NOX1, NOX2, NOX3 and NOX4 contains two haem groups which function as an electron carrier, and two binding sites for the co-enzymes FAD and NADPH. NOXs catalyse the NADPH-dependent reduction of oxygen to form superoxide [192]. Thus, although ROS is produced as a by-product of several catalytic activities, the presence of ROS producing enzymes such as NOXs suggests a role of ROS in promoting cellular functions.

### 5.2. ROS: Double-Edged Modulators of Endothelial Function

ROS production has a role in regulating EC proliferation, vascular permeability [193,194,195] shear stress-induced vasodilation, hypoxia signalling, autophagy, and pro-inflammatory activation [42,196] via the modulation of several pathways such as NF-κB, MAPK, PI3K-Akt and calcium signalling [197,198]. Accordingly, Colavitti and colleagues showed that activation of VEGFR2 by VEGF-A in porcine aortic ECs rapidly increases the levels of hydrogen peroxide and demonstrated that ROS promotes ERK1/2 activity since treatment with ROS scavengers reduce ERK1/2 phosphorylation [199].

A recent study has demonstrated that cigarette smoke extract (CSE) damages the pulmonary ECs permeability, observed in Chronic Obstructive Pulmonary Disease (COPD) via mROS-induced NF-κB signalling [198]. CSE impairs the mitochondrial membrane potential thus increasing ROS production and decreasing the endothelial mitochondrial content. The increased ROS levels promote IκBα phosphorylation and p65 NF-κB nuclear translocation [198]. NF-κB signalling activation increases ECs pro-inflammatory response, via the increased expression of PECAM-1, VCAM-1, the secretion of IL-6, IL-8 and VEGF-A [194,200]. Furthermore, NF-κB activation reduces ECs survival by increasing the autophagy pathway downstream of LC3 and Beclin-1 and decreases the expression of VE-Cadherin, compromising the endothelial barrier integrity [198].

Visfatin, an adipocytokine produced in visceral fat [201] and overexpressed in obesity and type-2 diabetes [202], has been reported to promote ECs pro-inflammatory response through the phosphorylation of p38 MAPK triggering PI3K and Akt activation and increasing NOX4 activity. ROS produced by NOX4 activity, increases the phosphorylation of IKK, in turn leading to the nuclear translocation of NF-κB, which enhances VCAM-1 and ICAM-1 expression, thus, promoting monocyte-endothelial cell adhesion [203].

ROS production by NOX4 regulates the metabolic reprogramming in EC exposed to disturbed flow which increases NOX4 expression, modulating HIF1α in a ROS-dependent mechanism [20]. HIF1α promotes cellular glycolysis (Figure 4) and increases the expression of pyruvate dehydrogenase kinase-1 (PDK-1) resulting in reduced conversion of pyruvate into Acetyl-CoA and lower mitochondrial activity. In addition to increased glycolysis, HIF1α promotes VEGF-A expression and secretion, enhancing the tube formation of HUVEC, in a process involving ROS production [204]. Interestingly, during reoxygenation following an ischemic event in the heart, the generation of elevated ROS levels in cardiac microvascular endothelial cells promotes the phosphorylation of ERK1/2, p38 and JNK leading to an increased expression and nuclear translocation of the transcription factor Egr-1 leading to cell death [205]. Thus, while controlled ROS production plays a physiological role in ECs, high level of ROS has a toxic effect in ECs and induces EC dysfunction, cell death and senescence [206,207,208]. Cellular senescence decreases proliferation and migration and ECs senescence could impair vascular processes such as angiogenesis, nutrient trafficking and vascular repair [209]. Accordingly, senescent ECs are pro-inflammatory and pro-thrombotic and have a reduced capacity to metabolise atherogenic lipids [22]. Increased ROS levels have been shown to increase vascular permeability [210], to damage the vessel’s responsiveness to hypoxia [196] and to promote pro-inflammatory pathways [211,212,213]. In oxidative stress conditions, O_2_^−^ reacts with NO, to produce the nitrogen reactive species peroxynitrite (ONOO^−^,), thus decreasing NO bioavailability and promoting ONOO^−^-mediated protein nitration and consequent EC dysfunction and death [214,215]. Consistent with the idea that ROS, and potentially EC ROS, contribute to cardiovascular disease, elevated ROS levels are detected in the heart, kidney and blood vessels in mouse models of vascular hypertension [216,217], in hyper-glycaemia [218,219], and in atherosclerosis [219].

## 6. Neuropilin-1 and ATP Binding Cassette Subfamily B Member 8 (ABCB8): Two Modulators of Mitochondrial Function in ECs

**Figure 5:**
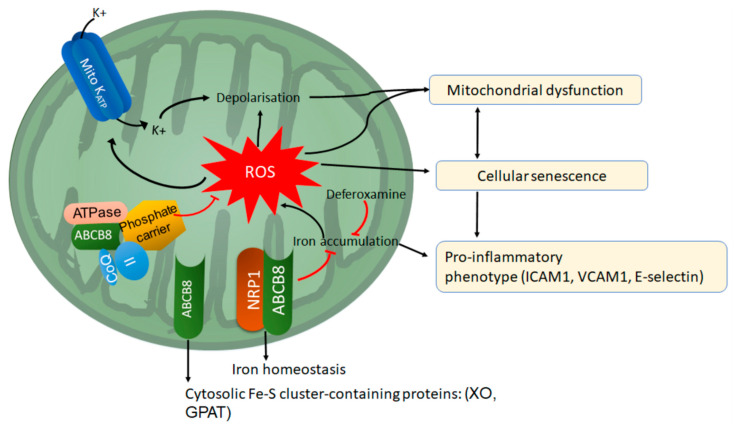
The NRP1-ABCB8 pathway: Schematic representation of the NRP1-ABCB8 pathway in regulating iron homeostasis, iron-dependent oxidative stress, mitochondrial function and cellular senescence.

Although mitochondrial metabolism and homeostasis are emerging as important regulators of endothelial function (see Section 4.3 and Section 4.4), the mechanisms regulating mitochondrial homeostasis, activity and dynamics in ECs are not completely understood. New evidence produced by our lab shows that NRP1 regulates mitochondrial content and function in ECs. NRP1 autonomously promotes mitochondrial homeostasis and function since NRP1 downregulation, but not VEGFR2 knockdown, reduces mitochondrial mass, mitochondrial membrane potential and inhibits mitochondrial dynamics by reducing Mitofusin-1 (MFN1) levels [135]. Consistently with a mitochondrial dysfunction phenotype, downregulation of NRP1 increases mROS in ECs and reduces the expression of the antioxidant enzymes SOD1 and SOD2 [135]. Furthermore, analysis of mutant *nrp1a*^sa1485^ zebrafish embryos lacking the expression of full-length Nrp1a show increased oxidative stress in the blood vessels compared to controls [135]. Mechanistically we found that a pool of NRP1 localises in the mitochondria where it interacts with the ATP Binding Cassette Subfamily B Member 8 (ABCB8) (see Section 6.2), protecting the mitochondria of ECs from iron-dependent oxidative stress and mitochondrial dysfunction which result in EC senescence [135]. Accordingly, NRP1 or ABCB8 downregulation in ECs induces accumulation of intracellular and mitochondrial iron, increasing iron-dependent mitochondrial ROS production. The consequent sustained iron-dependent oxidative stress in ABCB8- or NRP1-deficient cells, leads to reduced mitochondrial membrane potential and mitochondrial dysfunction, resulting in cellular senescence [135]. Accordingly, treatment of ABCB8- or NRP1-deficient ECs with the iron chelator Deferoxamine reduces oxidative stress, restores mitochondrial function and rescues the senescent phenotype [135] (Figure 5).

The discovery of an ABCB8-NRP1 pathway and its role in iron homeostasis and senescence in ECs highlights the importance of mitochondrial and iron homeostasis in ECs and prompts to further investigate the role of iron homeostasis and mitochondrial function in angiogenesis and vascular homeostasis. This finding highlights the importance of mitochondria in promoting endothelial function and agrees with previous evidence that mitochondria contribute to regulating cellular metabolism and biosynthetic pathways (see Section 4.3 and Section 4.4) in EC. Our observations also suggest that NRP1 could promote endothelial function and angiogenesis by modulating EC metabolism and mitochondria activity, in addition to modulating the VEGF-dependent and -independent signalling pathways (described in Section 3.4).

### 6.1. ABC Transporters

ABC transporters are transmembrane proteins found in all living organism that transport organic and inorganic molecules (e.g., xenobiotics, ions, metabolites, lipids, vitamins) across biological membranes [220]. In humans, 48 genes encoding ABC transporters have been identified and classified into seven subfamilies (A to G) according to their sequence homology [221]. Functional ABC transporters are constituted of four domains: Two transmembrane domains (TM) and two nucleotide-binding domains (NB) [222]. Each domain can be present in a single polypeptide or two domains can be fused in one longer protein [223]. Thus, the number of peptide subunits forming a functional ABC transporter varies across the ABC transporter family. The TMs bind the substrates and determine the substrate binding specificity while ATP hydrolysis occurs on the NB domains producing a conformational change in the NB and TM domains that allows substrate translocation across the lipid bilayer [224,225].

Four ABC transporters belonging to the subfamily B localise in mitochondria in mammalian cells. ABCB7, ABCB8 and ABCB10 localise in the inner membrane whilst ABCB6 is found in the outer membrane [226].

ABCB10 promotes haem biosynthesis in developing red blood and complexes with Mitoferrin-1, known to promote mitochondrial iron import [227,228]. Accordingly, murine erythroleukemic cells deficient for ABCB10 show decreased levels of Mitoferrin-1, increased iron uptake into mitochondria and reduced iron incorporation into haem [229]. Furthermore, downregulation of ABCB10 in zebrafish embryos reduces haemoglobinisation and erythrocytes number but embryos showed no accumulation of intermediate porphyrins or protoporphyrin IX, which are used in haemoglobin synthesis [229].

ABCB7 regulates Fe-S cluster biogenesis and loss of function mutation has been identified as the cause X-linked sideroblastic anaemia with ataxia [230]. ABCB7 downregulation in murine cell models of early stages of terminal red blood cell development reduces mitochondrial and cytoplasmic Fe-S cluster levels [231]. Recent studies have shown that ABCB7 regulates Fe-S cluster levels by transporting a glutathione/Fe-S cluster complex, in agreement with the reduced Fe-S cluster levels in the absence of ABCB7 [232,233]. ABCB7 has also been shown to promote mitochondrial Fe-S biogenesis and iron homeostasis by forming a multimeric complex with a dimeric ferrochelatase and ABCB10 homodimers by interacting with the NB of each transporter [231].

ABCB6 localises in mitochondria but is also found at the plasma membrane, Golgi, ER and lysosomes. Studies in erythroid cell lines and mice have shown that ABCB6 imports coproporhyrinogen III (CPgenIII), haem and protoporphyrin IX (PPIX) from the cytosol into the mitochondria [234,235].

### 6.2. ABCB8

ABCB8 is a 65 kDa protein located in the inner membrane of mitochondria [236] belonging to the Subfamily B of the ABC transporters. ABCB8 has been identified 20 years ago as the product of the ABCB8 gene and localises in the mitochondrial inner membrane [236]. As ABCB8 is a half-transporter, two ABCB8 monomers of 65kDa interact to form a full transporter [237]. Also, ABCB8 is present in multimeric mitochondrial complexes in the inner membrane and it interacts with succinate dehydrogenase, ATPase and the mitochondria phosphate carrier PIC to modulate the mitochondrial K_ATP_ channel activity [238,239] (Figure 5). A recent study has shown that ABCB8 interacts with the mitochondrial protein MITOK mediating ATP-dependent potassium currents and regulating mitochondrial volume and function [240]. Also, ABCB8 is a critical regulator of mitochondrial iron homeostasis and maturation of Fe-S cluster proteins in the cytoplasm. Accordingly, ABCB8 downregulation decreases the activity of xanthine oxidase (XO), aconitase and glutamate phosphoribosylpyrophosphate amidotransferase (GPAT), both containing Fe-S clusters [241]. Mouse mutants lacking ABCB8 expression in the heart have compromised systolic and diastolic function and show cardiomyopathy, fibrosis but no obvious signs of heart failure after 8 weeks from gene deletion [241]. Accordingly, the levels of ABCB8 expression decreases in the hearts of patients with end-stage cardiomyopathy [241]. Cardiomyocytes of ABCB8 knockouts show mitochondrial morphology defects such as reduced mitochondrial cristae and increased apoptosis. Furthermore, ABCB8 loss induces mitochondrial iron accumulation, oxidative stress, as well as decreasing the activity of cytosolic Fe-S cluster proteins [241]. Supporting a crucial role of ABCB8 in regulating iron homeostasis, ABCB8 mutants overexpressing ABCB8 are protected from cardiomyopathy induced by Doxorubicin, an anticancer drug known to induce iron-dependent oxidative stress in cardiomyocytes and cardiotoxicity [242]. Conversely, Doxorubicin treatment of mice with ABCB8 deletion in the heart exacerbated cardiomyopathy [242]. Besides its role in the cardiomyocyte protection, the role of ABCB8 in other cell types is poorly understood. Our recent study showing that ABCB8 regulates iron homeostasis also in ECs in a mechanism requiring NRP1 (Figure 5; see Section 6), suggests that ABCB8 could have a role in regulating EC-mediated processes such as angiogenesis and vascular homeostasis, mitochondrial homeostasis and EC metabolism.

Furthermore, our study suggests that ABCB8 function could be particularly relevant in age-related diseases characterised by increased oxidative stress or deregulation of iron metabolism (discussed in Section 7) such as atherosclerosis, vascular dementia, and Alzheimer’s disease (discussed in Section 8).

## 7. Iron Metabolism and Homeostasis

In biological systems, iron is found in iron-containing proteins such as haemoglobin, myoglobin, and enzymes containing Fe-S clusters. Iron is absorbed in the duodenum, where enterocytes absorb iron from the gut lumen. The absorbed iron forms the intracellular iron pool and it is then exported outside the enterocytes by the transmembrane transporter Ferroportin-1 (FPN1) localised in the basolateral membrane. The iron transported by ferroportin is then oxidised by ferroxidases into ferric iron (Fe^3+^) which binds to Transferrin in the interstitial fluids and the vasculature and is then distributed throughout the body [243]. In tissues and organs, the cellular internalisation of iron via a Clathrin-dependent endocytosis process requires the interaction of Transferrin with the Transferrin Receptor 1 (TfR1) which localises at the plasma membrane. Clathrin coated pit becomes a vesicle and fuses with early endosome whose acidic pH induces the release of Fe^3+^ from transferrin into the endosome [244]. The metalloreductases STEAP reduces Fe^3+^ to ferrous iron (Fe^2+^), which is then transported outside the endosomes by the Divalent Metal Transporter 1 (DMT1) to form the liable iron pool (LIP) [244]). LIP will then be used for the haem and iron–sulphur clusters biosynthetic pathways, stored as haem–ferritin or further exported outside of the cell by the transmembrane transporter Ferroportin-1 (FPN1) [245,246].

Thus, most of the iron is complexed to proteins and its amount in cells and tissues is tightly controlled via a homeostatic mechanism involving the peptide hormone hepcidin produced by hepatocytes. Hepcidin binds to FPN1 and induces its internalisation and degradation [247] in gut enterocytes, bone marrow hepatocytes, macrophages, bone marrow, splenic and mucosal cells [248], limiting dietary iron absorption, reducing cellular iron export and promoting iron storage in parenchymal tissues such as hepatocytes and islet cells of the pancreas [249]. Mutations in genes involved in systemic iron homeostasis such as Transferrin receptor 2, Hepcidin, Hemojuvelin and Ferroportin cause hereditary hemochromatosis (HH), characterised by systemic iron overload and tissue iron accumulation [250].

### 7.1. Role of Endothelial Cells in Iron Metabolism

Recently, ECs have emerged as key players of iron homeostasis. In response to iron, the specialised ECs in the liver sinusoids produce Bone Morphogenetic Protein-6 (BMP-6) which binds to its receptor in hepatocytes inducing the phosphorylation of SMAD1/5/8 and leading to the transcription and the expression of Hepcidin [251,252]. Accordingly, loss of BMP6 in ECs, but not in hepatocytes or macrophages, induces systemic tissue iron overload recapitulating the human HH phenotype in mice [253]. More recently, it has been shown that BMP2 collaborates with BMP6 to regulate iron homeostasis as double endothelial BMP6/BMP2 knockout mutants show similar hepcidin deficiency and tissue iron overload to single knockout mouse mutants [254]. Several, studies have shown the involvement of iron in the production of O_2_^−^ via the reduction of O_2_ by Fe-S clusters and free haem [255,256]. Also, iron can react with H_2_O_2_ to produce hydroxyl radicals (HO^•^) HO^•^ + HO^−^ [257,258]. The generation of ROS plays a role in the modulation of iron homeostasis as treatment with the mitochondria-targeted antioxidant compound MitoTEMPO prevents gene expression of BMP6 induced by iron [259], and conversely, the superoxide generation within the mitochondria induced by the mitochondria-targeted redox cycler MitoPQ [260] increases the expression of BMP6 in liver sinusoidal ECs and hepatocytes [259].

### 7.2. Effects of Iron Levels on Endothelial Function

In patients, HH is associated with vascular dysfunction [261,262,263] such as reduction of the endothelium-dependent dilation and increased intima-media thickness of the carotid artery and increased expression of soluble ICAM-1, soluble VCAM-1, VEGF and IL-6 [170,264,265], (Kartikasari, Georgiou et al. 2006). In mice Hfe-KO model of HH, endothelial-specific BMP2-KO, involved in the feedback mechanism of iron signalling, enhances the hemochromatosis phenotype [254], highlighting the role of iron in HH endothelial dysfunction. Taken together those studies suggest the tole of iron in the endothelial dysfunction. Accumulation of ionic iron induces the formation of redox-active iron pools able to catalyse the production of free radical via Fenton chemistry [266,267]. Accordingly, exposure of ECs to iron induces cellular oxidative stress and apoptosis, leading to a pro-inflammatory and pro-thrombotic response [264,268,269]. In ECs, treatments with exogenous iron induce the release of endothelial microparticles, whose release increases in activated ECs and this response is inhibited by iron chelation [170]. In addition, exogenous iron induces ROS generation in ECs, disrupting mitochondrial membrane potential (ΔΨ) which can be rescued by treatment with the iron chelator deferiprone [170]. Interestingly, treatment of ECs with 300 nM and 600 nM FeCl_3_ differentially affect mitochondrial respiration, with the lower dose increasing basal oxygen consumption and showing no effect on mitochondrial maximal respiration capacity, while the higher dose reducing both parameters which are rescued by iron chelation [170]. This difference could be due to the dose-dependent increasing cytotoxicity reflecting a decrease of the ROS detoxification functions together with an increase in apoptosis.

Iron overload in ECs increases the expression of pro-inflammatory markers such as VCAM-1, ICAM-1 and E-selectin [270] and decreases the activity of eNOS, impairing the endothelial-dependent relaxation of blood vessels [271]. Consistently with a detrimental effect of excessive iron towards endothelial function, we found that downregulation of the mitochondrial transporter ABCB8 in HUVECs and Human Microvascular ECs induces iron-dependent mitochondrial ROS production resulting in reduced mitochondrial activity and EC senescence [135]. Accordingly, treatment with the iron chelator deferoxamine restores mitochondrial function, reducing endothelial oxidative stress and rescuing the senescence phenotype in ABCB8-deficient ECs [135]. As NRP1 or ABCB8 downregulation similarly affect mitochondrial ROS, mitochondrial ΔΨ and EC senescence, and simultaneous downregulation of NRP1 and ABCB8 has no additive effects, NRP1 and ABCB8 promote iron homeostasis, mitochondrial function and EC senescence through a common pathway [135]. This increasing amount of evidence suggests that ECs have a central role in the regulation of systemic iron homeostasis and that iron-dependent endothelial dysfunction could contribute to vascular disease as well as non-vascular disease [272,273].

## 8. Role of EC Dysfunction in Pathology

### 8.1. Atherosclerosis

Cardiovascular disease is the main cause of death in Western countries and it is characterised by loss of endothelial function, consequent atherosclerosis and ultimately thrombosis and a cardiac, cerebral or vascular event.

Atherosclerosis is a chronic inflammatory vascular disease characterised by the presence of plaques formed by fat, cholesterol, calcified and fibrous material in the innermost layer of arterial vessels [274]. Risk factors such as low level of high-density lipoprotein (HDL), diabetes, smoking, obesity, advanced age and metabolic syndrome [275,276,277,278,279,280,281] contributes to vascular diseases. Whether iron plays a role in the onset and progression of atherosclerosis is controversial. The FeAST trial failed to demonstrate a beneficial effect of reducing body iron stores on cardiovascular disease mortality [282,283], while the Bruneck epidemiological study [284,285] and a study investigating the relationship between body iron stores and the risk of acute myocardial infarction [286] suggest that iron promotes atherosclerosis by mediating oxidative stress and lipid peroxidation. The discrepancy could be explained by methodological differences with the first study using serum ferritin as the indicator of the iron-load status, whose levels poorly reflect the tissue iron load involving non-transferrin bound iron [287].

#### 8.1.1. Role of Iron in Atherosclerosis

Human atherosclerotic vessels show increased expression of ferritin-encoding genes and intra-tissue iron deposit [288]. Treatment with exogenous iron of ApoE^−/−^ mouse mutants, an established mouse model of atherosclerosis, worsen the atherosclerotic phenotype inducing endothelial damage and dysfunction [289]. More recent evidence shows that ApoE^−/−^ mouse mutants carrying a mutation in the *Slc40a1* gene encoding FPN1 which is associated with Type IV HH in humans, show increased Non-Transferrin-Bound Iron (NTBI) and Liable Plasma Iron (LPI) levels. In these mouse mutants, both NTBI and LPI significantly correlate with an increased number and area of aortic atherosclerotic lesions and higher levels of oxidised LDLs [290]. Accordingly, double mutants showed increased endothelial dysfunction associated with increased vascular permeability, reduced NO bioavailability, and increased expression of pro-inflammatory adhesion molecules and cytokines [290]. In agreement, the elevated iron load in HH patients correlates with the high levels of circulating soluble adhesion molecules, lipid and protein oxidation, reduced NO and increased circulating inflammatory chemokines [290].

Electron paramagnetic resonance spectroscopy analysis of atherosclerotic carotid lesions shows that iron and copper accumulate in the endothelial monolayer and that iron levels further increase in advanced atherosclerotic lesions [291]. Although these studies overall suggest that iron has a role in vascular dysfunction and atherosclerosis, further studies are required to establish whether the accumulation of iron specifically in ECs contributes to the onset of the endothelial dysfunction and atherosclerosis. As treatment with exogenous iron of ApoE^−/−^ mice induces endothelial damage and dysfunction and reduces catalase and superoxide dismutase activity in the aorta [289], these results suggest that iron accumulation in ECs likely results in ROS-mediated endothelial damage. In agreement with this hypothesis, mitochondrial damage has been detected in atherosclerotic aortic vessels in human and mice [175]. Furthermore, ApoE^−/−^ mouse mutants with reduced expression of the mitochondrial antioxidant SOD2, develop a higher number of atherosclerotic lesions and show increased mitochondrial damage in the aortic tissue [175]. Future studies employing genetic tools to specifically delete in the endothelium the genes regulating iron homeostasis will be instrumental to define the role of endothelial iron homeostasis in vascular diseases.

#### 8.1.2. Atherosclerosis and Alzheimer’s Disease (AD)

Atherosclerosis of large and small cerebral vessels is associated with lower cognitive performance and increased risk for Alzheimer’s disease (AD) [292]. The human *APOE* gene exists as three polymorphic alleles (ε2, ε3 and ε4) with a frequency of 8.4%, 77.9% and 13.7% respectively in the world population [293]. A meta-analysis of data from 5930 AD patients and 8607 healthy controls shows that the risk of AD significantly increases in people with genotypes ε2/ε4; ε3/ε4; ε4/ε4 [293]. Accordingly, the frequency of the ε4 allele is dramatically increased to ~40% in patients with AD. AD is the most common form of dementia in 2019, affecting 1 in 14 people over 65 and 1 in 6 people over 80 and the disease’s incidence is predicted to triple by 2050 [294]. A recent clinical study has linked the genetic interaction of NRP1 and VEGFA with APOE-ε4 in the process of cognition [194]. They revealed that high expression of NRP1 correlates to a cognitive decline in the patients carrying the *APOE*-ε4 gene, whereas NRP1 is associated with a beneficial outcome for patients without the *APOE*-ε4 gene. Whether NRP1 modifies the risk associated with *APOE*-ε4 allele by increasing EC permeability [295] or whether compensatory mechanisms upregulate its expression to compensate for the endothelial dysfunction by promoting protective pathways such as the ABCB8/NRP1 pathway it is yet to be established.

### 8.2. Alzheimer’s Disease (AD)

AD pathology includes neuronal degeneration in the frontal cortex and hippocampus together with the enlargement of the cerebral ventricles. This causes loss of short-term memories, confusion and in late stages declining speech ability. The hallmarks of AD are the presence of extracellular amyloid plaques in the brain formed by β-amyloid-40 (Aβ40) and β-amyloid-42 (Aβ42) peptides which are the product of proteolytic cleavage of the Amyloid Precursor Peptide (APP) peptide fragments; the flame-shaped neurofibrillary tangles of the microtubule-binding protein tau in the lesions [294,296]. Although extensive research has shown that the expression of the proteases involved in APP cleavage increases in AD [297,298,299], it is now established that the impairment of peptide degradation also contributes to AD [300,301].

In addition to β-amyloid (Aβ) and tau pathobiology, cerebrovascular dysfunction and vascular pathology contribute to AD and increasing evidence strongly suggests that cerebrovascular dysfunction and vascular pathology is not merely a comorbidity but vascular damage and disfunction occurs either before [302] or in parallel of the accumulation of Aβ [302,303,304]. These studies suggest that endothelial dysfunction possibly initiates AD pathogenesis. Accordingly, 90% of AD patients show accumulation of insoluble Aβ in cortical and leptomeningeal arteries, arterioles and around the capillary walls [305,306], which leads to endothelial cell dysfunction and death [307].

#### 8.2.1. Blood–Brain Barrier Dysfunction and Alzheimer’s Disease

Endothelial cells in the brain form the blood–brain barrier (BBB), which tightly regulates solutes exchange between the lumen of blood vessels and the interstitium of the brain parenchyma. BBB breakdown causes accumulation in the brain parenchyma of blood-derived neurotoxic proteins such as fibrinogen, thrombin, haemoglobin, iron-containing hemosiderin and free iron, contributing to neurodegeneration [272,273]. In AD patients and AD animal models Aβ accumulation induces EC dysfunction and reduces the expression of tight junctional protein in brain ECs, thus resulting in decreased BBB tightness [308,309,310,311,312]. Recently, several groups reported that brain microvascular ECs produce amyloid-β peptide, suggesting a new endothelial-dependent pathway involved in Aβ deposition [313]. Interestingly treatment of human brain microvascular ECs with Cystatin C, a natural cysteine protease inhibitor preventing Aβ deposition in AD, reduces Aβ secretion promoting non-amyloidogenic processing of APP by increasing the SIRT1-dependent expression of the α-secretase ADAM10 [314]. This evidence further highlights the active role of ECs in AD pathology.

#### 8.2.2. The Role of Iron in Alzheimer’s Disease

The use of magnetic resonance imaging to analyse amyloid plaques in the brain of AD patients consistently shows focal iron deposition accompanying the plaques and that the extent of iron accumulation varies between brain regions [315]. Similarly, mouse models of AD display iron accumulation in amyloid plaques although lower compared to humans [315,316]. APP possess an iron-responsive element that increases its expression in the presence of iron [317], potentially establishing a vicious circle that further increases APP production. Since iron has been reported to interact with Aβ peptides, this interaction likely leads to the accumulation observed in the senile plaques and could be a key regulator of the formation of mature amyloid plaques [318,319,320,321,322]. Recent evidence in a zebrafish development model shows that cleaved secreted forms of APP, produced by neurons, accumulate at the embryonic vasculature in the venous vessels [323]. Secreted APP has been shown to bind and stabilise FPN1 at the plasma membrane [324,325], suggesting that in AD cleaved APP secreted from neurons binds to FPN1 at the basal surface of ECs in the BBB, promoting iron flux into the brain interstitium and contributing to dysregulate brain iron homeostasis [326].

Iron accumulation in ECs has a pro-inflammatory effect [270,271] and the secretion of pro-inflammatory cytokines TNF-α, IL-6 and IL-1β is increased in AD mice models and post-mortem patient’s brain [327]. Accordingly, AD patients present higher levels of soluble VCAM-1 and ICAM-1 in the plasma compared to control [328]. Soluble VCAM-1 levels correlate with advanced dementia suggesting that VCAM-1 could be used as a biomarker for cognitive decline in AD patients [329]. Iron accumulation is also associated with ROS production and in AD high levels of ROS are detected in neurons [330,331,332,333] and in the vascular wall [334]. Elevated ROS levels induce lipid peroxidation, resulting in the damage of cellular organelles such as the mitochondrial and contributing to neurons oxidative stress and endothelial dysfunction.

#### 8.2.3. Mitochondrial Dysfunction and Alzheimer’s Disease

Mitochondrial dysfunction occurs in AD and is associated with reduced dendritic mitochondrial content [335]. Whether impaired mitochondrial function contributes to the onset of AD or is a consequence of the pathological process is not completely understood. For instance, APP and Aβ colocalise in the mitochondria, reducing mitochondrial activity and respiration and change mitochondrial dynamics by promoting mitochondrial fission [336]. Furthermore, Aβ promotes mitochondrial-dependent and -independent apoptosis in brain microvascular ECs resulting in the release of cytochrome c from mitochondria and the activation of caspase 3, caspase 9 and caspase 12 [337,338,339]. On the other hand, treatment of HEK293 with the Complex I inhibitor Rotenone, which reduces ATP levels while increasing O_2_^−^ radicals and cytosolic ROS levels, or with Complex III inhibitor Antimycin, increases levels of secreted Aβ expression. Treatment with ROS scavengers significantly reduces the Rotenone-induced Aβ expression indicating that mitochondrial ROS enhances amyloidogenesis [340]. Accordingly, cybrid lines in which the mtDNA derives from AD patients show more increased Aβ levels compared to cybrid lines containing the mtDNA from healthy age-matched controls [341,342]. However, in vivo studies in mouse models of AD genetically modified through different approaches to also have mitochondrial dysfunction, show mixed results. Some studies found that mitochondrial dysfunction increases Aβ plaque deposition [343] and others showed a reduced number of Aβ plaques [344,345]. Together, these studies indicate that a complex scenario exists in which mitochondria function regulates different mechanisms which likely differentially affect functions in different cell types (i.e., neurons and ECs) and further studies aimed at understanding the cell-specific contribution of mitochondrial dysfunction to cell function are required to better understand the role of mitochondria in the progression of AD. As EC dysfunction is a common aspect to neurodegenerative diseases such as Alzheimer’s disease, Parkinson’s disease, Huntington’s disease, amyotrophic lateral sclerosis, multiple sclerosis, HIV-1-associated dementia and chronic traumatic encephalopathy [346], future research focussing on understanding the signalling and metabolic pathways promoting EC homeostasis and those inducing EC dysfunction will provide key basic knowledge to develop treatments to prevent or treat these diseases.

## 9. Conclusions

ECs are an essential component of the vascular system as they regulate vascular function and consequently blood supply to the organs of all vertebrates. Since Napoleone Ferrara and his colleagues at Genentech isolated and cloned VEGF-A in 1989, cardiovascular research has profoundly increased the knowledge by which cytokines and signalling pathways regulates EC growth and the formation of new blood vessels. This research has allowed developing drugs to treat diseases characterised by pathological angiogenesis or increased vascular permeability. The recent discoveries that modulation of metabolic pathways regulates EC function, has highlighted the crosstalk between metabolism and signalling pathways and point to EC metabolism as a promising research area to identify new therapeutic targets to regulate EC function. As seen throughout this review, iron homeostasis and ROS signalling are emerging as additional key players in regulating physiological EC function. The growing evidence that iron accumulation occurs in ECs in many vascular pathologies, such as atherosclerosis, or in diseases characterised by vascular dysfunction such as neurodegenerative diseases, shows the homeostatic nature of the endothelium that maintains iron flux into the surrounding tissue and that therefore is likely to undergo iron overload following local or systemic iron variations. The double-edged role of ROS on EC function as a physiological signalling molecule or as a damaging by-product of cellular metabolism and redox reactions highlights the mitochondria as a cellular crossroad where metabolism and signalling co-operate to modulate EC function. Although the mechanism by which mitochondria contributes to EC function is not completely understood, mitochondria are a potential therapeutic target for treating diseases characterised by aberrant angiogenesis such as retinopathies and cancer growth, as shown by the reduction in angiogenesis when anaplerosis is impaired. Endothelial-specific modulation of mitochondrial function and metabolism or of iron homeostasis in mice models of cardiovascular or neurodegenerative diseases (i.e., atherosclerosis, AD, Parkinson’s disease and multiple sclerosis) will shed a light on the contribution of EC metabolism in vascular homeostasis and vascular disease and could highlight potential new therapies to treat these pathologies.
